# Is Work Time Control Good for Innovation? A Two-Stage Study to Verify the Mediating and Moderating Processes

**DOI:** 10.3389/fpsyg.2022.821441

**Published:** 2022-02-01

**Authors:** Xiao Pan, Xiaokang Zhao, Huali Shen

**Affiliations:** ^1^Glorious Sun School of Business and Management, Donghua University, Shanghai, China; ^2^School of Business, Nantong Institute of Technology, Nantong, China; ^3^School of Business, Pingxiang University, Pingxiang, China

**Keywords:** working time control, knowledge employee, job demands-resources model (JD-R model), self-regulation theory, innovation

## Abstract

As a part of job resources, work time control is essential for innovation. We examine how work time control impacts knowledge employees’ innovation in the workplace. A two-stage study was conducted to verify the mediating and moderating processes. In Study 1, adopting the job demands–resources model as a theoretical framework, we conducted a laboratory test to find the relation between work time control, job engagement, job burnout, and innovation, and verified the path between work time control and innovation. In Study 2, drawing on the job demands–resources model verified by Study 1 and self-regulation theory, it is proposed that during the psychological process in the workplace, job engagement plays a mediating role, and the vocational delay of gratification plays a moderating role between work time control and innovation. A total of 254 knowledge employees from diverse organizations participated in the survey study. After taking demographic variables, job demands, and neuroticism as control variables, the results showed that job engagement would mediate the relationship between work time control and innovation. A higher level of delay of gratification buffered the effect of a higher level of work time control on innovation. All these findings verified and expanded knowledge on work time control and innovation literature, showing that work time control is important for innovation. Based on Chinese cultural background, managers should offer employees the opportunity to conduct self-control training and encourage them with great freedom to foster employee innovation.

## Introduction

In recent years, with the prevalence of overtime work such as “white + black,” “5 + 2,” or “996” in China, the debate over whether work time control matters for innovation has become much more severe and urgent. Managers in Chinese enterprises are trying to increase employees’ opportunities to innovate by prolonging working hours. In this domain, some research has shown that employees who have sufficient freedom during their work process have a greater chance of coming up with unconventional ideas and combining novel work procedures (Volmer et al., 2012). However, is work time control good for innovation remains to be verified and expanded theoretically and empirically. A more comprehensive understanding of the psychological mechanism between work time control and innovation needs to be explored.

The McKinsey (2007) global survey has reported that it is essential for managers to find the right people engaged in innovation and align them for innovation. We address the above issues by integrating the job demands–resources model and self-regulation theory in a novel way. The job demands–resources model provides a macrostructure to define how work time control may contribute to innovation (Bakker et al., 2014). Hence, job engagement and job burnout take the mediating roles between work time control and innovation. In addition, according to the self-regulation theory, the process may be strengthened by the vocational delay of gratification. It has been found that when employees are aware of the expected profit from behavioral outcomes, they will tend to pursue long-term benefits, even though they are less motivated (Fishbach and Trope, 2005; Liu and Yu, 2017).

This research initiative has contributed to work time control and innovation literature and practice. First, we conducted a laboratory test to identify the relation between work time control, job engagement, job burnout, and innovation and verified the path between work time control and innovation based on the job demands–resources model. Derived from this model, job engagement and burnout are identified as core psychological processes. Second, we expanded current theory in this field by integrating the job demands–resources model with self-regulation theory in a novel way to construct a more comprehensive model, seeking the relationship between work time control and innovation under the moderation of vocational delay of gratification. Third, based on the laboratory test, we conducted a survey that obtains data to verify the sophisticated statistical model, including mediation and moderation. Finally, we informed practitioners about how to cultivate knowledge employees in a new way to foster innovation in the workplace. In the following part of this article, after combining the job demands–resources model with self-regulation theory, we hypothesized the interplay among work time control, vocational delay of gratification, job engagement, job burnout, and innovation. A two-stage study that combines laboratory tests with empirical research is conducted to test these theoretical proposals.

## Theory and Hypotheses

### Work Time Control and Job-Related Results

Aimed to achieve good performance, employees need to obtain job resources, which facilitate employees to achieve goals in the workplace (Bakker et al., 2014). As a part of job resources, job control, involving the control of work progress, skill discretion, decision-making power, participation in decision-making, and predictability have already been proven to be closely related to job attitudes and performance (Ala-Mursula, 2002). We paid attention to work time control because it is closely related to management and execution (Amabile et al., 2002). Working time control refers to the possibility that employees control the duration, location, and distribution of working time, that is, autonomous control of working time (Ala-Mursula, 2006). It also means the opportunity for individuals to determine their work schedule (Wall et al., 1996; Beckers et al., 2012).

Self-determination theory points out that as one of the core requirements of the diffusion of intrinsic motivation, autonomy has a significant and positive relationship with job motivation and job satisfaction by effectively coping with relatively difficult situations (e.g., high workloads or long hours) (Deci and Ryan, 2008). According to these theories, since work time control is one of the forms of work autonomy, we can assume that work time control can promote work motivation, increase job satisfaction, contribute to employee retention, improve work efficiency, and innovation (Hoskins, 2014; Madrid and Patterson, 2020).

Individuals have different abilities to control their working hours. A relatively higher level of work time control positively impacts employees’ physiology, including improving sleep quality, reducing depression symptoms, disease, and tired feelings (Tucker et al., 2015; Virtanen et al., 2021). A higher level of work time control also will significantly improve job satisfaction and keep work and life balance, which can even affect employees’ retirement age (Virtanen et al., 2014). All the elements above have a positive impact on employee mobility, which is beneficial for organizations to attract and retain talents (Barrett and Holme, 2018). On the other hand, a low level of working time control is often associated with psychological stress, increasing the risk of sleep disorders and cardiovascular morbidity, and reducing individual health perception (Ala-Mursula, 2002; Salo et al., 2014). However, other research has shown that the implementation of work time methods applied in organizations has no impact on psychosocial characteristics or other work-related outcomes (Nijp et al., 2016; Karhula et al., 2020). Under most circumstances, employees with a high level of work time control have more tendency to choose longer continuous leisure time even at the expense of adequate recovery or even workplace function to make work schedules that are contrary to ergonomic principles (Kecklund et al., 2008; Barrett and Holme, 2018). The contradictory research results indicate that the complex correlation between work time control and job-related results are still worthy and interesting. Research on the relationship between work time control and innovation is relatively scarce and needs further exploration.

### Job Demands–Resources

The job demands–resources model is a theoretical framework to comprehend the motivation process and the health-impaired process to performance in the workplace (Bakker and Demerouti, 2017). Job resources, including physical and psychological job elements (e.g., job control, feedback, and organizational support), facilitate job performance. Job demands play a role on the contrary (Demerouti et al., 2014). On the motivation process, it is found that when employees are faced with challenges at work, work resources inspire job engagement, which translates into a willingness and effort to perform well according to the organization’s requirements (Wall et al., 1996). Job engagement, namely, a positive work-related state of mind, characterized by vigor, dedication, and absorption, has been attracting attention as a critical factor in improving work productivity (Bakker et al., 2014). In other words, employees tend to devote themselves to work when they obtain relatively higher job resources. From the perspective of resource conservation theory, one type of resource helps individuals obtain other types of resources to maximize the total amount of resources (Hobfoll, 1989). For individuals, pleasure and energy play a key role in balancing between depletion and gains of psychological processes, which provide the basis for greater well-being and performance (Bakker and Leiter, 2010). Employees with high levels of job engagement resulting from sufficient job resources have more chance to expand their range of thoughts and integrate diverse ideas effectively, which is expected to contribute to better job performance (Fredrickson, 2001). The facilitation of the generation and integration of ideas are also helpful in innovation. As an antecedent to innovativeness, job engagement will mediate the relation between job resources and proactive behavior, which creates a basis for innovation (Haque, 2009). Focusing on the impaired process, based on conservation of resource theory, employees will show symptoms of burnout when they lack job resources at work (Lee and Ashforth, 1996). Job burnout is often referred to mental and physical exhaustion, namely, a state of weariness, including exhaustion, cynicism, and professional efficacy. Some prior researches characterize burnout as three opposite dimensions of work engagement. However, later research verified that burnout and engagement are negatively correlated and independent rather than two opposite poles (Maslach and Leiter, 1997; Russell and Carroll, 1999). When the individuals who lack job resources still tend to work as usual, two options are open (Hockey, 1997). One is a strain coping mode, which maintains target performance at the expense of increasing compensatory costs on psychology and physiology. The other is a passive coping mode, which means lowering target performance. According to the theories of health promotion and maintenance, the employees have a low level of work time control, which means a lack of external environment resources. Under such circumstances, employees will disengage from work and reduce motivation to inspire self-protection mechanisms, which may have a greater chance to reduce future frustrations of not obtaining stated goals (Demerouti et al., 2001). Hence, on the contrary, job burnout may be regarded as an inhibitor of innovation.

**FIGURE 1:**
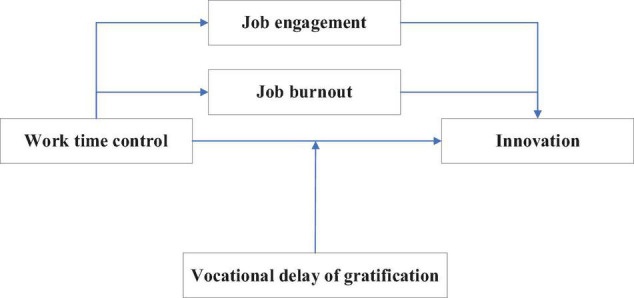
Model of the predicted relationships between work time control and innovation, mediated by job engagement and job burnout and moderated by vocational delay of gratification.

As a sound theoretical framework, the job demands–resources model provides mechanisms to understand the relationship between work time control, job engagement, job burnout, and innovation. The mediating roles of job engagement and burnout between work time control and innovation are also examined (see Figure 1 for a model). For knowledge employees in contemporary organizations worldwide, the demand for autonomy is a universal trend. Based on that reason, we expand the mediating effect of job engagement and job burnout found in Western samples to Asian samples. Drawing on the above reasoning, two hypotheses are proposed:

**Hypothesis 1:** Job engagement will play a mediating role in the relationship between work time control and innovation, on the basis that work time control will have a positive effect on job engagement, which will have a positive effect on innovation.

**Hypothesis 2:** Job burnout will play a mediating role in the relationship between work time control and innovation, on the basis that work time control will have a negative effect on job burnout, which will have a negative effect on innovation.

### Self-Regulation Theory

Job resources not only can act directly at work but also can help individuals gain more personal resources. The flexible use of job resources and personal resources help to promote innovation in return. Some researchers point out that besides job resources, personal resources may help adjust threats, buffer the relation between demands and adverse outcomes, and predict positive outcomes effectively (Xanthopoulou et al., 2007; Hobfoll, 2011). According to a self-regulation theory, self-regulation can adjust both situational and individual factors and has been widely applied to managerial work, employee socialization, and employee performance (Strakowski et al., 2009). This theory emphasizes individuals’ ability to guide goal-directed activities according to their standards. To conduct acts of volition, individuals need to use personal resources to obtain some form of energy during the process (Muraven et al., 1998). This process is in accordance with employees’ active innovation process and therefore offers the basis for employees’ innovation (De Stobbeleir et al., 2011). Delay of gratification, namely, individuals’ ability to forego immediate gratification to attain a more valuable outcome later on during the work, is a kind of self-regulation that includes planning, controlling, and waiting. It belongs to personal resources and is closely related to job satisfaction, career development, and job performance. For the model developed here, vocational delay of gratification is proposed to moderate work time control and innovation (He et al., 2015). Based on the job demands–resources model, this study verifies the relationship between work time control, job engagement, job burnout, and innovation. The moderating role of vocational delay of gratification between work time control and innovation is also examined (Figure 1 for a model).

Drawing on the above reasoning, the third hypothesis is proposed:

**Hypothesis 3:** Vocational delay of gratification will play a moderating role in the relationship between work time control and innovation, which means among knowledge employees with a lower level of delay of gratification, the impact of work time control on innovation will be significantly stronger than among those with a higher level of delay of gratification.

### Overview of the Studies

To explore the association between work time control and innovation and how the psychological process unfolded under the influence of vocational delay of gratification, a two-stage study including a laboratory test (i.e., Study 1) and an empirical survey (i.e., Study 2) was conducted to verify the theoretical model. In Study 1, adopting the job demands–resources model as a theoretical framework, we conducted an experience to test correlation among work time control, job engagement, job burnout, and innovation and try to verify the path between work time control and innovation. To further validate and enrich the model, in Study 2, we conducted the survey empirically to test the mediator (i.e., job engagement) and the moderator (i.e., vocational delay of gratification) between work time control and innovation. This study was approved by the Blinded Ethics Committee, and written informed consent was obtained from all participants.

## Study 1

### Sample and Procedures

Aimed to test correlation among work time control, job engagement, job burnout, and innovation, and try to verify the path between work time control and innovation, Study 1 was conducted in the laboratory. Before the experiment, we recruited 76 college students to fill in the revised Eysenck Personality Questionnaire. In order to control the neuroticism level, 17 of them with extreme scores were excluded, the others were recruited to participate in the formal experiment based on their scores.

All the participants were divided into an experimental group (*N* = 29) and a control group (*N* = 30) randomly. They were required to participate in a comprehensive simulation experiment by FlexSim software in the laboratory. The participants in the experimental group have the freedom to control their work time, whereas the experimental controller ultimately arranges the others in the control group from 8:00 a.m. to 6:00 p.m. After completing the FlexSim program, the participants were required to complete questionnaires to self-estimate job engagement, job burnout, and innovation. The age of the participants was around 20 and 62.7% was female.

### Measures

#### Neuroticism

A 12-item scale revised from the revised Eysenck Personality Questionnaire (EPQ-R; Eysenck et al., 1985; Hughes and Parkes, 2007) was adapted to measure neuroticism (e.g., Are you a worrier?), and its applicability has already been examined under the Chinese culture (Qian et al., 2000). Scores on the dichotomous item responses (yes = 1, no = 0) were summed. Low scores indicate a low level of neuroticism. Mean item scores were analyzed and α = 0.71.

#### Job Engagement

A 9-item scale (UWES-9) was adapted to measure job engagement. It consists of three subscales, including vigor (e.g., At my work, I feel bursting with energy), dedication (e.g., I am enthusiastic about my job), and absorption (e.g., I feel happy when I am working intensely) (Schaufeli et al., 2006). A 5-point Likert scale ranging from 1 (not at all) to 5 (a great extent) was adapted. Low scores on all three dimensions indicate a low level of job engagement. Mean item scores were analyzed with α = 0.93.

#### Job Burnout

A 16-item scale (MBI-GS) was adapted to measure job burnout. It consists of three subscales, including exhaustion (e.g., I feel my work was really tiring), cynicism (e.g., I feel my job was boring), and professional efficacy (e.g., I feel I am making a useful contribution to the company) (Schaufeli et al., 1996). A 5-point Likert scale ranging from 1 (not at all) to 5 (a great extent) was used. Since all professional efficacy items are scored reversibly, low scores on professional efficacy and high scores on exhaustion and cynicism mean a high level of job burnout. Mean item scores were analyzed with α = 0.92.

#### Innovation

A 3-item scale developed by Janssen (2000) was adapted to assess innovation. It consists of three subscales, including idea generation (e.g., I will create new ideas for difficult issues), idea promotion (e.g., I will mobilize support for innovative ideas), idea realization (e.g., I will transform innovative ideas into useful applications). A 5-point Likert scale ranging from 1 (never) to 5 (many times) was used. We require the individuals to self-estimate the extent to which they generated new ideas, promoted new ideas, or realized new ideas in their jobs. Low scores on all three dimensions indicate low innovation. Mean item scores were analyzed with α = 0.94.

### Analytical Strategy

After the normal distribution of measures was examined, an independent sample *t*-test was conducted to inspect the difference in job engagement, job burnout, and innovation between the experimental and control groups. Then we used PROCESS to make a regression analysis to verify the mediation between work time control, job engagement, job burnout, and innovation. Robust estimation based on bootstrapping techniques was used simultaneously (Hayes, 2013).

### Study 1 Results

There were 29 subjects in the experimental group and 30 subjects in the control group. Table 1 showed the means, standard deviations, and correlations of the variables. The group was highly correlated to job engagement (*r* = 0.27, *p* < 0.05) and innovation (*r* = 0.57, *p* < 0.01), but not correlated to gender (*r* = −0.13, *p* > 0.05) and job burnout (*r* = 0.1, *p* > 0.05). According to results of the independent sample *t*-test, there is a significant difference in job engagement (*p* < 0.05) and innovation (*p* < 0.01) between the experimental group and the control group. However, there is no significant difference in job burnout (*p* > 0.05) between the experimental group and the control group.

**TABLE 1:** Means, standard deviations, and Pearson correlations among study variables.

Variables	Mean	SD	1	2	3	4	5
1. Gender^a^	0.37	0.49	1.00				
2. Group^b^	0.49	0.50	–0.13	1.00			
3. JE	29.44	6.77	−0.27*	0.29*	1.00		
4. JB	33.29	9.12	0.04	0.10	−0.55**	1.00	
5. Innovation	28.88	5.10	–0.25	0.57**	0.65**	−0.34**	1.00

*p < 0.05; **p < 0.01.

*^a^Gender: 0 = female, 1 = male.*

*^b^Group: 0 = control group, 1 = experimental group.*

*JE, job engagement; JB, job burnout.*

Hypothesis 1 and 2 indicated that job engagement and job burnout would mediate the relationship between work time control and innovation separately. According to the job-demands resource model, there are two paths, including the gain and loss paths. The gain path is the motivational process. The regression analysis demonstrated that work time control had a highly significant and positive correlation with job engagement (*b* = 3.95, SE = 1.70, *p* < 0.05) and innovation (*b* = 4.67, SE = 0.96, *p* < 0.01). Job engagement also was positively related to innovation (*b* = 0.33, SE = 0.85, *p* < 0.01). Moreover, a significant indirect effect of work time control on innovation *via* job engagement was verified (*b* = 1.29, *p* < 0.01), which indicated the presence of a partial mediation process. The loss path is the health-impaired process. The regression analysis shows that work time control has no significant indirect effect on innovation *via* job burnout. Hypothesis 1 was supported, and Hypothesis 2 was not supported.

## Study 2

### Sample and Procedures

Based on Study 1, a survey was carried out in China to verify the hypotheses further. This survey was conducted both online and underline to explore knowledge employees’ perceptions of work time control and innovation. As the invitation stated, all the participants were requested to fill in their demographic information, self-estimated levels of work time control, self-estimated levels of job engagement, and innovative behaviors carried out in their workplaces. In addition, after completing the questionnaires, all the participants were encouraged to share the questionnaires with colleagues and friends for the sake of expanding the coverage of the data.

A total of 326 individuals were recruited to complete the questionnaires. Since this research took knowledge employees as the primary research objects, 72 cases were deleted due to incorrect research objects or incomplete data for all variables measured. The response rate was 77.91%. The mean age of 254 participants was 31.48 (SD = 6.31), and 90.55% of them had undertaken university studies. The gender of these participants was 59.06% female and 61% of the participants were married. Around 48.4% of the participants had at least one child. The mean organizational tenure of participants was 7.39 years (SD = 7.61). The participants consisted of ordinary employees (55.1%), grassroots managers (24%), middle managers (15.4%), and senior managers (5.5%).

### Measures

#### Work Time Control

A 4-item scale was adapted to assess levels of work time control (Wall et al., 1996). Individuals were required to self-estimate the levels of work time control they had in the workplace (e.g., Can you decide on the order in which you do things?). A 5-point Likert scale ranging from 1 (not at all) to 5 (a great extent) was adapted. Low scores indicated a low level of work time control. Mean item scores were analyzed, α = 0.90.

#### Job Engagement

A 9-items scale (UWES-9) was adapted to measure job engagement as in Study 1.

#### Vocational Delay of Gratification

An 8-item scale (VDGQ) was adapted to assess the delay of gratification (Liu et al., 2007). A 5-point Likert scale ranging from 1 (strongly disagree) to 5 (strongly agree) was adapted to self-estimate employees’ levels of vocational delay of gratification (e.g., It is not a problem to start my career as an ordinary clerk as long as there is a promotion possibility). Low scores represented a low level of vocational delay of gratification. Mean item scores were analyzed, α = 0.73.

#### Innovation

A 3-item scale developed by Janssen was adapted to measure innovation as in Study 1.

#### Control Variables

In previous studies, van der Hulst (2003), van der Hulst et al. (2006) criticized that potential confounder factors (e.g., demographic variables and personality) had not been included in the work hours and work-related outcomes literature. This study included gender, age, marital status, education level, and the number of children as control variables as these demographics might correlate with innovation. Based on the prevailing circumstance, higher job demands significantly correlated with work-related results, and neuroticism may lead to negative work behavior. It was also important to take neuroticism and job demands as control variables, which help avoid possible confounding effects among work time control, job engagement, and innovation (Janssen et al., 2004; Podsakoff et al., 2012).

##### Job Demands

A 5-item scale revised from JCQ was adapted to self-estimate psychological job demands (e.g., I need to complete my work within a very tight time) (Karasek et al., 1998). A 5-point Likert scale ranging from 1 (strongly disagree) to 5 (strongly agree) was used. Low scores indicate low job demands. Mean item scores were analyzed, α = 0.75.

##### Neuroticism

A 12-item scale revised from EPQ-R was adapted to measure neuroticism as in Study 1 (Eysenck et al., 1985; Hughes and Parkes, 2007).

### Analytical Strategy

A two-stage analytical strategy was conducted in the study. To test the validity and robustness of the measurement model, a series of confirmatory factor analyses were conducted before regression analysis. The concrete analytical strategy is as follows.

After the normal distribution test, a four-factor model comprising the variables (i.e., work time control, job engagement, vocational delay of gratification, and innovation) was tested based on hypotheses. Then, a chi-squared difference test was adapted to compare the single-factor model with the four-factor model to determine whether the common method variance may affect subsequent analyses or not (Podsakoff et al., 2012). Regression analysis was carried out by PROCESS to verify the mediation of job engagement and moderation of vocational delay of gratification between work time control and innovation. Robust estimation based on bootstrapping techniques was used simultaneously (Hayes, 2013).

### Study 2 Results

According to the confirmatory factor analysis, the four-factor model, including work time control, job engagement, delay of gratification, and innovation (χ^2^ = 336.6, df = 129, RMSEA = 0.08, SRMR = 0.075, CFI = 0.926, TLI = 0.912), demonstrated a significantly better goodness-of-fit than the single-factor model (χ^2^ = 1281.3, df = 135, RMSEA = 0.183, SRMR = 0.129, CFI = 0.592, TLI = 0.537) and was also significantly better than the single-factor model [Δχ^2^(df) = 744.7(6), *p* < 0.01]. The above results not only supported the robustness of the hypothesis-based measurement model, but also showed that the common method variance had been controlled.

Table 2 showed the means, standard deviations, and correlations of the variables. The demographic information, including age, gender, marital status, number of children, educational level, and organizational tenure, was not related to innovation. Job demands and neuroticism were included in the subsequent analyses as control variables.

**TABLE 2:** Means, standard deviations, and Pearson correlations among study variables (*N* = 254).

Variables	Mean	SD	1	2	3	4	5	6	7	8	9	10	11	12
1. Gender^a^	0.41	0.49	1.00											
2. Age	31.48	6.30	0.12	1.00										
3. Marital status^b^	1.61	0.49	–0.02	0.54**	1.00									
4. Number of children	0.61	0.71	–0.11	0.54**	0.68**	1.00								
5. Education level	3.37	0.77	0.00	−0.34**	−0.26**	−0.45**	1.00							
6. Organizational tenure	7.39	7.61	0.17**	0.84**	0.44**	0.47**	−0.40**	1.00						
7. Job demands	3.47	0.82	–0.10	0.04	0.04	0.08	–0.11	0.03	1.00					
8. Neuroticism	16.49	2.41	0.10	0.03	–0.01	0.01	–0.02	0.02	0.03	1.00				
9. WTC	3.65	0.82	−0.16*	0.06	0.11	0.09	–0.08	0.03	0.239**	0.18**	1.00			
10. JE	3.54	0.72	0.03	0.15*	0.05	0.01	0.01	0.08	0.193**	0.19**	0.55**	1.00		
11. VDG	2.83	0.44	0.06	–0.01	–0.12	−0.14*	0.140*	–0.04	0.252**	0.02	0.22**	0.50**	1.00	
12. Innovation	3.66	0.73	0.03	0.10	0.04	–0.02	0.11	0.07	0.230**	0.205**	0.44**	0.65**	0.52**	1.00

*p < 0.05; **p < 0.01.

*^a^Gender: 0 = female, 1 = male.*

*^b^Marital status: 1 = unmarried, 2 = married.*

*WTC, work time control; JE, job engagement; VDG, vocational delay of gratification.*

Based on Study 1, job engagement was verified to play a mediating role in the relation between work time control and innovation (Hypothesis 1). In Study 2, Model 1 demonstrated that work time control had a highly significant and positive correlation with job engagement (*b* = 0.453, SE = 0.049, *p* < 0.01) and innovation (*b* = 0.337, SE = 0.052, *p* < 0.01). Job engagement also had a highly significant and positive correlation with innovation (*b* = 0.652, SE = 0.048, *p* < 0.01). Moreover, a significant indirect effect of work time control on innovation *via* job engagement was verified (*b* = 0.258, *p* < 0.01), which indicated the existence of a partial mediation process. Hypothesis 1 was supported by the results shown in Table 3.

**TABLE 3:** Mediation and moderated-mediation in Model 1 and Model 2.

Variable	Model 1: mediation		Model 2: mod-mediation	
	Job engagement	Innovation	Job engagement	Innovation
Intercept	1.17 (0.32)**	0.68 (0.29)	1.17 (0.32)**	−1.43 (0.72)
Direct effects				
Job demands	0.06 (0.05)	0.09 (0.04)*	0.06 (0.05)	0.05 (0.04)
Neuroticism	0.03 (0.01)	0.02 (0.01)	0.03 (0.02)	0.03 (0.01)
WTC	0.45 (0.05)**	0.08 (0.05)	0.45 (0.05)**	0.48 (0.19)*
VDG				0.92 (0.25)**
JE		0.57 (0.06)**		0.42 (0.06)**
Indirect effect [Bootstrap = 10,000]	0.26 [0.17, 0.36]*			
*F* (df1, df2)	38.00 (3, 250)**	50.00 (4, 249)**		
*R*^2^ model	0.56**	0.67**		
Interactive term			−0.13 (0.06)*	
WTC × VDG				
*F* (df1, df2)			38.00 (3, 250)**	41.75 (6, 247)**
R^2^ model			0.56**	0.71**

*p < 0.05; **p < 0.01.

**FIGURE 2:**
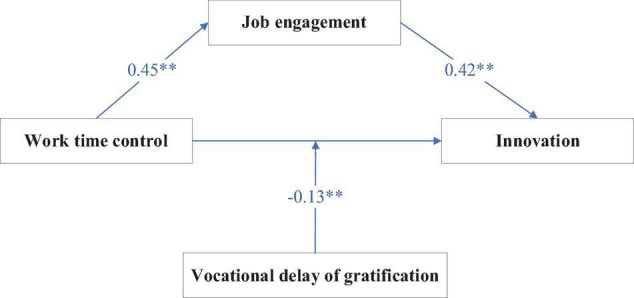
Moderated-mediation model with the variables studied. **p < 0.01.

**FIGURE 3:**
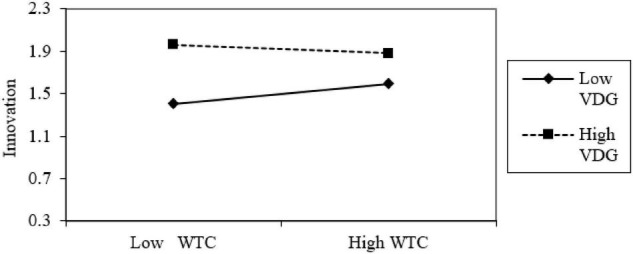
Interaction effect between work time control and vocational delay of gratification on innovation.

Hypothesis 3 indicated that vocational delay of gratification would moderate the relation between work time control and innovation. According to the moderated-mediation analysis of Model 2 in Table 3, work time control had a highly significant and positive correlation with job engagement (*b* = 0.45, SE = 0.05, *p* < 0.01). Job engagement also had a highly significant and positive correlation with innovation (*b* = 0.42, SE = 0.06, *p* < 0.01). Interaction term between work time control and vocational delay of gratification had a negative relation with innovation (*b* = −0.13, SE = 0.06, *p* < 0.01). The moderated-mediation model is shown in Figure 2. Figure 3 showed that work time control had a significant and positive relationship with innovation when the vocational delay of gratification was at a low level (−1 SD *b* = −0.44, *p* < 0.01). However, work time control had a non-significant relation with the innovation when vocational delay of gratification was at a high level (+1 SD *b* = 0.44, *p* > 0.05). Hypothesis 3 was supported by the results.

## Discussion

A two-stage study was conducted to verify the mediating effect of job engagement (job burnout) and the differential moderating effect of vocational delay of gratification on the work time control–innovation relationship among Chinese knowledge employees. As predicted, Hypotheses 1 and Hypotheses 3 in this study were supported. This result verified the predictions derived from the job demands–resources model and self-regulation theory. In concrete terms, bivariate correlations in Table 2 showed that the innovation had a significant relation with job demands, neuroticism, work time control, job engagement, and vocational delay of gratification. In the later regression analyses, job engagement was demonstrated to mediate between work time control and innovation, and vocational delay of gratification was demonstrated to moderate work time control–innovation relationship in a weakening way. Knowledge employees with low levels of vocational delay of gratification are more likely to generate, promote, and realize new ideas when they have high levels of work time control. These findings in this study have implications not only for theory, but also for practice. In most circumstances, knowledge employees in the organizations are eager to transcend their roles and show more engagement in activities to gain more job resources (e.g., work time control) as well as personal resources (e.g., vocational delay of gratification).

Hypothesis 2 in this study was not supported. This result showed that work time control had no relation with job burnout, and job burnout was not able to play a mediating role in the relation between work time control and innovation. The result is consistent with Karhula et al.’s (2020) research. According to a previous research, burnout is caused by a continuing strain resulting from a lack of consistency between job demands and job resources (Maslach et al., 2001). Employees with a relatively low level of work time control are more likely to get a feeling of fatigue, which needs time to accumulate and ultimately leads to job burnout. In addition, because of the characteristics of the knowledge employees, when they have low levels of work time control, they are more likely to choose the passive coping mode rather than the strain coping mode, which means downward adjustment of performance targets rather than maintaining target performance at the expense of increasing compensatory costs on psychology and physiology. The relatively high levels of authority offer knowledge employees the opportunity to have a more relaxed space for loafing behaviors (e.g., cyberloafing behaviors) (Davenport, 2013). In other words, knowledge employees with low levels of work time control might have a big chance to get entrapped in a hostile, vicious spiral. They are more likely not prone to strive for changes in such situations. In this way, their feelings of burnout will not increase either (Bakker et al., 2003).

### Theoretical Implications

This study contributes to work time control and innovation literature from a theoretical stance. It includes work time control, job engagement, job burnout, vocational delay of gratification, and innovation as variables and investigates them in one study. Based on the job demands–resources model, the present findings verified the path between work time control and innovation and integrated the job demands–resources model and self-regulation theory in a novel way. In concrete terms, as a part of job resources, work time control offers knowledge employees the opportunities to meet their basic psychological needs of autonomy. This kind of satisfaction is closely related to job engagement, which coordinates with the motivational process’s gain path. This finding is in accordance with prior findings that underscore the critical influence of work time control on the employees’ innovation from diverse organizations (Madrid and Patterson, 2020). During the motivation process, job engagement is a positive, fulfilling, and work-related state of mind, including vigor, dedication, and absorption (Schaufeli et al., 2002), which motivates knowledge employees to concentrate on and devote their energy to increased job satisfaction, higher personal initiative, and innovation motivation (Salanova et al., 2010). In addition, according to the conservation of resources theory, vocational delay of gratification, as a kind of personal resource, may replenish job resources to adjust to valuable psychological processes for innovation (Hobfoll, 1989). In this study, we focus on knowledge employees as they are the main body of innovation, and all the variables mentioned above are the prominent characteristics.

### Practical Implications

From a practical stance, the current finding is that increased control over work time is helpful for the stimulation of innovation, especially for the knowledge employees with a relatively low level of vocational delay of gratification. During the motivation processes, a relatively higher level of self-control on work time is more conducive to the innovative processes because job autonomy is a predictive strength on employee behavior, which is consistent with earlier research (Janssen and Van Yperen, 2004). Based on the Chinese cultural background, these findings have an important implication for managers in realistic conditions. During the human resource process, managers should get aware of employees’ levels of work time control and vocational delay of gratification to make a work plan. It also makes significant sense to measure the correlative factors constantly and find gaps in the work systems and processes. It is of great importance to offer employees the opportunity to conduct self-control training to enhance their work-related autonomy when needed. After prescribing a set of strategic goals, managers should encourage and allow employees great freedom instead of controlling them in the context of the goals (Tuan and Venkatesh, 2010). It is not only beneficial to promote employees’ innovation but also beneficial for organization training and exploitation, conducive to long-term development. The current study focuses on the individual level, but still provides a basis for the multilevel research on innovation.

Due to the differences in working time schedules and cultural backgrounds, further study is needed to explore whether the results in this study can be generalized to other cultures besides China. On the one hand, some governments across Europe have introduced new rules designed to encourage more participation-driven flexibility during the working hours. In recent years, Dutch law has given each employee the right to decide flexible working hours (such as the start and end of work). Similar laws have been introduced in Britain and Germany to improve employees’ working time flexibility (Nijp et al., 2016). Different from the normal flexible working arrangement in western countries, most employees in China have fixed working hours, sometimes overtime work. On the other hand, influenced by Confucian culture, China is a typical country with high power distance (Hofstede, 2001). When faced with top-down and high-intensity working time control, Chinese employees with strong collectivist preferences tend to be easier to comply with the arrangement and adapt to the working schedule than employees in other countries worldwide.

### Limitations and Future Directions

This study enriches the job demands–resource model and self-regulation theory, explores the relationship between work time control and innovation further, and provides inspirations for training and exploitation in organizations. However, there are some limitations. First, the sample range can be expanded in future studies to improve the reliability of data analysis. Second, self-report data in this study may result in an overestimation of the strength of associations between predictor and outcome variables (Podsakoff et al., 2003). Third, the cross-sectional design of the study ignores variables’ dynamic nature. These variables, including work time control, job engagement, job burnout, and innovation, fluctuate highly over days or even weeks. Longitudinal design (diary study), adapted in recent research, will be more suitable in some variables’ estimation (Bolger and Laurenceau, 2013; Madrid et al., 2014). Last, this study was carried out in a sample of knowledge employees with professional knowledge and skills. When generalizing these results to a broader population, we should pay attention to individuals’ characteristics.

## Conclusion

In recent years, with the prevalence of overtime work, the problem of work time control has become much more severe and urgent. Studies on working time control have already been published in some international academic journals. In conclusion, working time control is an emerging and essential topic in academia and modern work. Relatively speaking, there is little research on work time control and its impact on knowledge employee’s innovation in China. A two-stage study was conducted to expand understanding of the relationship between work time control and innovative behavior. Study 1 showed job engagement work as an explanatory mechanism between work time control and innovation. Study 2 showed vocational delay of gratification work as a boundary condition between work time control and innovation. We trust these findings will enrich the theory on job resources and innovation and foster organizational effectiveness.

## Data Availability Statement

The raw data supporting the conclusions of this article will be made available by the authors, without undue reservation.

## Ethics Statement

This study was approved by the Donghua University Ethics Committee and written informed consent was obtained from all participants. The patients/participants provided their written informed consent to participate in this study.

## Author Contributions

XP was involved in data collection, data cleaning, theory building, writing, and data analysis. XZ helped in theory building, editing, project administration, and supervision. HS was responsible for data collection and editing. All authors contributed to the article and approved the submitted version.

## Conflict of Interest

The authors declare that the research was conducted in the absence of any commercial or financial relationships that could be construed as a potential conflict of interest.

## Publisher’s Note

All claims expressed in this article are solely those of the authors and do not necessarily represent those of their affiliated organizations, or those of the publisher, the editors and the reviewers. Any product that may be evaluated in this article, or claim that may be made by its manufacturer, is not guaranteed or endorsed by the publisher.
